# A framework for improved classroom communication in the South African schooling context

**DOI:** 10.1016/j.heliyon.2021.e06382

**Published:** 2021-03-08

**Authors:** Brenton Fredericks, Gregory Alexander

**Affiliations:** a Department of Communication Studies, Central University of Technology, South Africa; b Department of Post Graduate Studies in Education, Psychology of Education, Central University of Technology, South Africa

**Keywords:** Communication, Knowledge transfer, Schooling context

## Abstract

The way in which information is conveyed should contribute positively to learners' overall learning experience and development. Knowledge transfer is a communication activity in the classroom and should therefore be effective. In the study reported in this article, an exploratory mixed method approach was applied to collect data regarding educators' perceptions of communication in South African schools. The results reveal the ways in which effective communication improves learner achievement, and conversely, how ineffective communication leads to a communication breakdown, misunderstanding and poor learner achievement. An educator communication framework is proposed to improve classroom communication in South African schools.

## 1 Introduction

As learners in the current information age are no longer perceived as passive recipients of knowledge, they are more inclined to question what they are being taught than in the past. Questioning is regarded by educationists as a powerful thinking processing skill which is grounded in the cognitive functioning of critical thinking, creative thinking and problem solving (Cuccio-Schirripa and Steiner, 2000). Therefore, the way information is disseminated should be effective for learners to understand why they require certain skills. Educators must be adept at conveying information in a constructive manner to ensure that content knowledge is learnt, and skills acquired. Transfer of knowledge occurs when information is conveyed from one person to another or among a group of people. Therefore, an educator deliberately transfers skills, information and knowledge to learners (Howells and Roberts, 2000). Welch and Welch (2008) and Smith (2015) point out that transferring knowledge is not as simple as it seems because communication barriers, such as poor listening, the lack of attention, interest, distractions, differences in viewpoints, hearing problems and speech difficulties are always present, which could negatively affect the transfer of knowledge. This problem is exacerbated not only by a revised curriculum in South African schools, but also by learners who are difficult. This implies learners who are disruptive in class, question figures of authority and show a disinterest in their schoolwork. Classroom communication factors that could impede learner performance have been investigated to establish whether learner performance is negatively affected or not (Osakwe, 2009), but the dynamics of the new generation of learners and the discrepancies due to the mismatch in educational strategies have not been extensively explored.

Communication forms an integral part of the learning experience that could enhance learner achievement. However, in contemporary classroom spaces there seem to be a communication breakdown between learners and educators (Asrar et al., 2018). This has also been the experience of the authors in South Africa. Since communication is not a one-dimensional process but interactional, learners should be actively involved in their own learning. Additionally, for the process of teaching and learning in the classroom to be meaningful, skilful and effective communication is required (Fashiku, 2017). Thus, to be effective, communication between educators and learners in the classroom should allow learners to master educational outcomes and acquire skills that they will need in the future. By the very nature of their work, educators are required to convey meaningful information to learners. The extent to which they are successful in their teaching depends on their ability to communicate effectively (Prozetsky, 2000). At this level, communication could be one-on-one or in a group. Educators communicate with learners through various means which could be verbal, non-verbal or writing (Duta et al., 2014). Therefore, the success of a communication activity and whether educators impart information effectively relies on their ability to convey information clearly and succinctly.

The aim of the study on which the article draws was to identify discrepancies and ways of communication in the changing educational environment. An awareness of communication factors that affect new generations of learners could significantly improve communication between educators and their learners.

## 2 Theoretical framework

The deliberations for this paper are based on social constructivism as theoretical framework. Within this context, the way information is transferred between educators and learners seems to be derived from communities of understanding rather than individual learners functioning in isolation from the classroom context. Through the lens of social constructivism, knowledge is constructed through individual interactions with the environment and all that is at the disposal of learners, whether they operate on an individual and/or group level (Mpisi, 2010). Learners are therefore affected by one another in a learning context that is characterised by mutual understanding and healthy social interaction (Cottone, 2007; Sohel, 2010). Knowledge is not an attribute of an individual, but is mutually constructed through active learning opportunities, shared experiences and various meaning-making realities learners are exposed to in their daily classroom encounters (Alexander & November, 2010). This process, we contend, forms the basis for effective communication, especially learning environments that are becoming increasingly diverse in nature. Furthermore, Jackson et al. (2006) argue that social constructivists view reality as constructed through human activity; that knowledge is a human product which is socially and culturally constructed; and that learning is a social process that occurs when individuals are engaged in social activity. Therefore, effective practices negotiated in mutually beneficiary classroom settings could assist in creating deeper understanding and richer social interactions between educators and learners. Our contention is that educators need to rethink how they communicate with their learners. Learners are no longer viewed as non-responsive participants to educational content as they were in the past. The question is not only about what they are taught, but also the relevance of curriculum content used in the teaching process. The way in which information is transferred should promote learners' overall learning experience in a constructive manner. Knowledge transfer is a communication activity in the classroom and should as such be utilised as an effective language and pedagogical tool.

## 3 Communication factors that influence learner achievement

Factors that could either contribute to effective and meaningful communication between educators and learners or impede the learning process resulting in a communication breakdown are relevant and warrant attention. Verbal communication, non-verbal communication, verbal aggression, communication apprehension, immediacy and teaching styles are all relevant factors discussed in the following sections.

### 3.1 Verbal communication

Educators and learners alike should be proficient in the Language of Learning and Teaching (LOLT) to be able to communicate effectively. In South Africa many learners receive instruction in English - a language that is not their mother tongue (Uys et al., 2007). These authors express concern that not all educators pay attention to the four basic language skills of reading, writing, speaking and listening. Similarly, Van der Poll and Van der Poll (2007) argue that learners need to obtain a level of mastery in the language in which they receive instruction to be able to understand the learning content in other subjects. They claim that understanding the LOLT is a requirement for learners if they wish to be academically successful. If learners are not proficient in the medium of instruction (Legotla et al., 2002) they might underachieve. In South Africa, learners are taught in either a first, second or third additional language. These learners have difficulty comprehending learning content as it is presented in a language that is not their mother tongue. In support of Legotla et al. (2002), Howie (2005) states that learners in urban areas who speak the language of instruction and have had considerable exposure to that language have a greater chance at succeeding than learners in rural areas. In South Africa, English has become the medium of instruction preferred by many parents, learners and institutions of learning. Jones (1990) as cited in McLauchlin (2007) points out that learners who have trouble understanding the LOLT are penalized because they are not able to express themselves adequately during spoken and written communication activities. Areas of potential conflict relating to verbal communication include dialect differences, especially grammar, morphology, vocabulary semantics and discussion modes. Learners who speak any non-standard dialect are often perceived as uneducated or less intelligent (Bennett, 2007).

Wahyuni (2017) in his study on the power of both verbal and non-verbal communication in leaning, notes the following regarding verbal communication. Educators who are proficient in the medium of instruction contribute significantly towards improving learner performance because they appear to have the skill in transferring information in both the written and spoken word in an effective manner. They conclude by stating and confirming the views in this study of the authors that good and effective communication skills enhance teaching and learning and ultimately learner performance.

Therefore, social interaction requires the use of language in a context that manifests modalities of effective communication. People have an innate ability to put their understanding into words – this essentially marks an advance in both understanding and development and as such have critical implications for teachers, who are regarded as the facilitators of learning (Eggen and Kauchak, 2014). Verbal communication therefore poses a threat to the educational experience, especially if learners are not proficient in the LOLT. This argument seems to be true for educators as well for those who have difficulty expressing themselves in English.

### 3.2 Non-verbal communication

According to Bambaeerooi and Shokrpour (2017), non-verbal communication is more refined in a way that it carries more depth during communication, more so in a teaching and learning environment. Therefore, its importance should not be underrated during teaching and learning as well as the impact that it could have on learner achievement. Bennett (2007) concurs that non-verbal communication finds expression in messages sent by individuals through unconscious body movements, such as facial expressions, gestures (kinesics), the unconscious use of personal space (proxemics) and unconscious physical touching (haptic). During communication activities, especially in classrooms, educators use gestures to impart information regarding subject matter, feelings and opinions. These gestures happen consciously or unconsciously but contribute significantly towards the overall communication activity. According to Raman and Sharma (2007), the verbal aspect of communication conveys only approximately 35% of the message, whereas the non-verbal component carries about 65% of the message. Therefore, the importance of non-verbal communication during classroom communication activities should not be underestimated as it could contribute either positively or negatively to the learning experience. An understanding of the impact that gestures have on people's perceptions of others will lead to informed and conscious choices regarding the use of certain gestures. Educators who work in multicultural and diverse classrooms should be aware of the importance of gestures during interaction with learners. In this regard, Ryan (1995) exhorts that appropriate actions should be selected to convey the intended message so that it can be interpreted correctly. Le Roux (2002) recommends that educators in South Africa remain perceptive to non-verbal messages that are conveyed in multicultural classroom environments. Gestures also assist learners in distinguishing between facts and opinions, while clarifying the meaning of terms. Learners even model certain gestures learnt from their educators (Orton, 2007). Similarly, Baringer and McCrosky (2000) state that non-verbal immediacy in the classroom results in both learners and educators achieving their goal, that is, enabling educators to convey their message effectively and learners to grasp learning content. In this way educational outcomes are achieved. Therefore, one may deduce that if educators and learners are aware of the important relationship between non-verbal communication and learning, they could identify ways of improving learner performance.

### 3.3 Verbal aggression

Miscommunication between educators and learners could be the cause of instances of verbal aggression reported in schools in South Africa (Prins, 2009). Incongruities of this nature negatively affect the relationship between educators and learners and the learning experience. De Wet (2006) claims that both educators and learners make themselves guilty of verbal aggression. Hassandri et al., (2007) discuss the negative consequences of verbal aggression and mention that it could instill feelings of low self-worth and result in the development of low self-concept. Verbal aggression thus defeats the purpose of transferring knowledge, values and a skill set that encourages good citizenship. In addition, the new generation of learners seem to be less willing to accept verbal abuse from educators.

Learners who have been subjected to this form of abuse should focus on developing positive interpersonal relations and, in extreme cases, seek professional assistance. Myburgh and Poggenpoel (2009) state that learners who have been affected should focus on developing good interpersonal relations. Educators and learners should also improve their listening skills for increased comprehension of what is being said instead of listening only to provide a response. Uludag (2013) investigated the influence aggression has on learner achievement and have found that influences academic performance negatively. His study reveals that even students who display aggressive behaviour obtain lower scores than learners who have less aggressive tendencies. This finding confirms prior studies findings that aggressive behaviour displayed by either learners or educators negatively impact learner performance.

### 3.4 Communication apprehension

When educators or learners have a fear of communicating with one another they experience communication apprehension (Robinson, 2007). Educators and learners could experience this apprehension in a classroom setting as the LOLT may not be their mother tongue. Learners experience anxiety or fear of embarrassment should they respond to educators' questions or participate in classroom discussions. Miller and Nadler (2009) explain that learners' levels of anxiety and fear can be so high before communicating that they fail to communicate effectively with educators or fellow learners. Studies conducted by Neer (1990) and Robinson (2007) have confirmed that learners experience communication apprehension in educational environments and educators should help learners to gain confidence and participate in classroom discussions. It is not only learners who are affected, educators also experience communication apprehension, especially new educators entering the field. Roby's (2009) research in this regard reveals that educators are apprehensive about communicating in groups, public speaking and in some instances one-on-one dialogues. A fear of communicating publicly could adversely affect their performance in class and have a negative impact on learner achievement. More importantly, miscommunication can occur as a result of communication apprehension and influence how learners interpret the messages they are sending.

### 3.5 Immediacy

It is difficult for learning to take place in a hostile learning environment. Good interpersonal relationships should exist between educators and learners to promote the acquisition of knowledge and skills. Robinson (2007) asserts that good relationships between educators and learners create a positive learning environment and reduce the physical and psychological distance between educators and learners. In so doing, learning becomes a pleasant experience which educators should strive towards achieving. Bainbridge-Frymier and Houser (2000) believe that effective communication skills are important to achieve good teaching practices, referential skill, ego support and conflict management. Referential skill implies the process of explaining and clarifying, which is significant in the context of learning and teaching and remains key to learners achieving educational outcomes. Educators provide direction in developing good interpersonal relationships with learners and in this regard should lead by example as learners often model their behaviours. Hawking (2005) supports this notion that educators play a crucial role in fostering a classroom environment that is conducive to teaching and learning. It should be an environment where learners have the freedom to express their opinions frankly and honestly. Attentive listening, coupled with good interpersonal relations in our experience with the new generation of learners, has shown that learners have a voice and that they want educators to listen to their viewpoints. According to Guffey and Loewy (2011), listening is an intricate process that needs communicators to be attentive not only to their surroundings, but also to the senders who are conveying information in a way. Educators should have effective listening skills to understand problems that learners experience during the transfer of knowledge. Similarly, learners should have good listening skills because they also need to understand the messages communicated to them by their educators. Noteworthy, in this regard, is the argument by Berko et al. (2010) that listening is enhanced if the listener is not prone to prejudgement, understands that communication is a two-way process and that both senders and receivers have a responsibility to listen attentively during communication activities.

Furthermore, a distinction needs to be drawn between immediacy and interpersonal skills which are both essential requisites for effective communication. Immediacy refers to good interpersonal relationships, whereas interpersonal skills refer to soft skills that people require to be successful and that are important during communication activities (Young, 2005). Interpersonal skills are needed during interactions especially when striving towards achieving a specific goal (Osakwe, 2009). It is also necessary to demonstrate appropriate social behaviours that are appropriate to specific contexts such as a classroom environment and learners should know this (Osakwe, 2009). Ultimately, amiable and caring relationships will develop between educators and learners and, in so doing, foster good social, emotional and academic functioning of learners (Bergsman et al., 2013).

Akif Sözer (2019) investigated educator immediacy based on how learners perceived educator behaviour. The results reveal that learners who have good interpersonal relationships with learners and demonstrate positive behaviour in classes are more effective than educators who do not have sound relationships with learners. Noteworthy of this study is that immediacy was frequently observed by way of non-verbal immediacy. This is implying that educators were observed through their actions such as gestures, eye contact and even smiling. Educators who frequently displayed a friendly attitude towards learners were deemed to be more efficient and the results of these learners were better than learners, who perceived their educators in a negative light.

### 3.6 Teaching as an interactive process

Teaching is a communicative activity in which the transfer of information or knowledge takes place. The way messages are conveyed determines the success or failure of an educational activity (Osakwe, 2009). Teaching styles should be varied to reach as many learners as possible. Mji and Magato (2006) contend that varying teaching styles accommodate most learners. In addition, Duta et al. (2014) state that educators' viewpoints regarding a topic affects the teaching style/s they employ in the learning environment. They explain that educators have a personal, pedagogical or interactional stance. The personal viewpoint focusses on how educators and learners perceive their roles in an educational environment. Educators should not be viewed as knowledge banks responsible for educating learners, nor should learners be recipients of knowledge. Rather, learners should be engaged in the learning process and educators should facilitate the learning process. The pedagogical viewpoint refers specifically to how educators view their responsibility as facilitators and how learners view their responsibilities as learners in an educational context. The interactional viewpoint was of primary importance to this study, describing how educators interact in the teaching environment and how learners perform their tasks in the classroom.

As the authors of this paper, we argue that an understanding of communication differences extends beyond the mere desire for constructive pedagogical relationships and enabling classroom interactions. These engagements promote healthy teacher-learner relationships which in turn embed effective communicative practices that seek the continuous negotiation and renegotiation of identities between the various participants (educators and learners) involved. Ultimately, teaching, learning and being able to function in a group context, such as multicultural educational settings mean being aware of differences and essentially, embracing diversity. An interactional approach is therefore tantamount to the acquisition of a skill set that will equip learners to think critically and solve problems, especially in a multicultural schooling context. In addition, the advent of the computer age has changed the face of education. Lehman and Dufrene (2011) state that constant improvements in technology have broadened communication preferences for organisations such as schools. However, technology should enhance communication in the classroom and not replace the important role of the educator. The use of technology enhances classroom communication and enriches learning experiences for learners (Lim and Morris, 2009), especially in well-resourced schools. Yet, there are schools that lack resources, especially those in rural areas.

## 4 Materials and methods

A discussion is given regarding the research process for the study, reported in this paper.

### 4.1 Research paradigm

This study formed part of a broader study that investigated the influence of macro (the whole school) and micro (classroom) communication factors on learner achievement in the Mangaung municipal area of South Africa. An interpretative paradigm was used because we wanted to gain a deeper understanding of how educators feel about communication in the classroom (Cohen et al., 2011), more especially because it is where they spend most of their working lives. A mixed-method research approach was used to explore educators' perceptions of communication as a factor in learner achievement, in addition to comprehending the complexities involved in knowledge transfer from educators to learners. We followed Schumacher and McMillan's (2006) recommendation that quantitative data should first be collected before qualitative data is utilised since the former can be used to authenticate qualitative findings.

### 4.2 Data gathering tools

The data-gathering tools were a questionnaire and interviews to address the research question (Bertram and Christiansen, 2019). A combination of these tools was used to ensure the reliability and validity of the research findings. A School Communication Effectiveness Questionnaire (SCEQ) was used as a data-gathering instrument to collect quantitative data and interviews were conducted to collect qualitative data. A statistician assisted with the design and layout of the questionnaire and to ensure that the research questions were addressed. The items in the questionnaire included: rank order questions, a 5-point Likert scale (ranging from “not at all” to “to a very large extent) and opened-ended questions. A pilot study was conducted to identify gaps in the questionnaire. It was piloted at three schools to see whether any ambiguities or duplications were present in the questionnaire. The pilot study revealed that Question 9.4 was duplicated. The researchers corrected the questionnaire before distributing it to the broader sample.

The respondents were requested through interviews with educators to express their views regarding the effectiveness of communication in their classrooms. The questions included educators' opinions about communication during interaction with learners, characteristics of effective communicators, the role of language proficiency, the impact of different teaching styles on effectively conveying information to learners and the role of communication apprehension and verbal aggression in the classroom. The interview guide was developed from information obtained from the literature and when the SCEQ was designed.

### 4.3 Study population and selection of respondents

The population for this study included educators from primary and high schools in the Mangaung area of the Free State province in South Africa. Twenty schools were selected from all five educational districts (Motheo, Xhariep, Thabo Mofutenyane, Lejweleputswa and Fezile Dabi) to provide a comprehensive account of how educators in the different districts experience communication in their classrooms. A degree of criterion sampling was used because the researcher selected 10 schools that performed academically well and 10 schools that were regarded as dysfunctional. The schools were selected from all five districts (4 schools from each district) based on results obtained from the Department of Education.

### 4.4 Data analysis

Data emerging from the SCEQ data gathering tool was analysed using a statistical programme known as the Statistical Package for the Social Sciences (SPSS). In addition, a rotated factor matrix and reliability analysis of communication factors that could influence learner achievement was used. A rotated factor matrix refers to investigating variables that were formerly not clustered into one group (Cohen et al., 2011). The variables grouped together in this study include verbal communication, non-verbal communication, verbal aggression, communication apprehension and teaching as an interactive process and have been grouped as micro communication factors that influence learner achievement. The aim of the exploratory factor matrix (EFA) was to summarise the interrelationships among variables in a concise, but conceptually accurate manner. EFA is utilised when an investigator wants to find out how the number of factors impacts variables and what would be the best approach to evaluate which variables can be grouped into a specific category (DeCoster, 1998). This procedure involved three steps, namely, preparation of the matrix and variables being analysed, extracting initial factors and rotating the factors to a terminal solution. This was done to ensure the reliability of the research findings.

Table 1 reveals the purpose of using an exploratory factor analysis to indicate the extent to which micro communication factors influence learner achievement in the classroom. Factor 1 is labelled Classroom Communication (a = .841) with loadings ranging from 0.896 to 0.795. From this information the researchers have concluded that communication will influence learner achievement, especially how effective educators teach and communicate in the classroom.

**Table 1:** Rotated factor matrix and reliability analysis of micro communication factors on learner achievement.

Extent to which the following micro communication factors influence learner achievement	Factor 1
1 (a = .842) Factor Classroom communication	
Verbal Communication	0.896
Non-verbal Communication	0.814
Verbal aggression	0.795
Communication apprehension	0.861
Immediacy	0.897
Teaching as an interactive process	0.851

The reliability of items in the questionnaire was computed using Cronbach's Alpha-coefficient. Cohen et al. (2011) explain that Cronbach's Alpha coefficient, ‘provides a coefficient of inter item correlations. What it does is calculate the average of all possible split-half reliability coefficients. It is a measure of the internal consistency among items and is used for multi-item scales’ (Cohen et al., 2011:640) (see Table 2).

**Table 2:** Cronbach's Alpha-coefficient.

Item in questionnaire	Cronbach's Alpha coefficient
Micro-communication	0.86

A = 0.7.

According to Bryman and Cramer the reliability level is acceptable if it is calculated at approximately 0.8 (1990). In this study, which forms part of a much bigger study (communication information from education stakeholders, methods of communication macro communication factors and micro communication) the overall Cronbach Alpha coefficient is 0.7, which indicates that the findings are reliable.

The transcriptions from the interviews were analysed using Cohen et al.'s (2011) recommendations: firstly, generating natural units of meaning from the interviews held with educators, and thereafter classifying and ordering these units of meaning into specific themes. The next step was to structure narratives to describe the interview contents and finally interpreting the data obtained from interviews. Thereafter, the data obtained from the questionnaires were synthesised using descriptive statistics that described respondents' views about effective communication in the classroom.

### 4.5 Ethical considerations

Permission to conduct this investigation was obtained from the Free State Department of Education, the ethics committee of the Central University of Technology, as well as principals and educators. Respondents signed informed consent forms in which they were assured of their anonymity. All schools that participated in the study have not been named.

## 5 Results and discussion

The findings indicate that communication factors, such as verbal communication, non-verbal communication, verbal aggression, communication apprehension, immediacy and teaching styles influence the transfer of knowledge. Based on these findings a framework was developed to provide educators with insight into how effective communication can improve learner achievement. Knowledge of these factors could enrich teaching practices.

Firstly, it was found that verbal communication does influence learner achievement. Most respondents (91%) indicated that verbal communication, especially fluency in the LOLT, influences learner achievement to a large extent. This finding validates our view that educators should be fluent in the LOLT. It also affirms Legotla et al., (2002) view that language proficiency is important during the transfer of knowledge in a classroom.

The following verbatim responses confirm that verbal communication is important during the transfer of information from educator to learner:

*Of course, but it could influence learner achievement positively or negatively. If you have a good command of the LOLT, learner achievement is often good. On the other hand, if the educator does not have a good command of the language, learner performance could be negatively affected. Clear, concise messages need to be conveyed to learners to grasp learning content (Resp D).*

*It will definitely influence learner achievement. Communication plays such an integral part of the learning experience (Resp F)*

The second factor that needs attention is non-verbal communication. The majority of respondents agreed (63%) that non-verbal communication in the classroom influences learner achievement to a large extent. Orton's (2007) view that educators use non-verbal communication to augment verbal communication was therefore validated. However, it was found that not all educators realised the importance of gestures during knowledge transfer. Responses such as “*I think it is important” (Resp A)* and *“Most times I am unaware of my gestures” (Resp C*) show that, despite the majority of participants indicating that non-verbal communication is important, the magnitude of its relevance was not understood by all.

The third factor that needs to be considered is verbal aggression. Interestingly, 49% of the respondents agreed that verbal aggression, such as educators using belittling remarks in the classroom, influences learner achievement. However, 51% felt that this factor does not influence learner achievement. Contrary to this finding, researchers such as Sullivan (2000) and Tevan (2001) found that verbal aggression does limit freedom of expression in the classroom, and ultimately learner performance. This finding is also contrary to the view that verbal aggression obstructs learner performance. We anticipated that a larger percentage of respondents would have agreed that verbal aggression influences learner achievement. Thus, we recommend that educators are enlightened about the negative consequences of verbal aggression. In so doing they could change their views about verbal aggression and refrain from verbally abusing learners in the classroom where this behaviour is practiced. During the interviewing it also be became apparent that numerous educators were not familiar with the concept of verbal aggression, as shown in some of their responses:

*I will be frank. Sometimes it is the only language they understand. I need to get a syllabus done and my HoD wants the work to be done. (Resp E)*

*We cannot say anything to the learners these days then it is a problem. (Resp B)*

The fourth factor to consider is communication apprehension. Fifty-four percent of the respondents indicated that communication apprehension influences learner achievement to a very large extent. The remaining 46% of respondents agreed that this factor influences learner achievement to a small extent or not at all. The research of Chesebro et al. (1992) indicates that communication apprehension influences learner performance negatively. It was expected that a higher percentage of respondents would agree that communication could influence learner achievement. Initially, during the interview, educators did not fully comprehend what communication apprehension entailed but once the term was explained to them, they were keen to elaborate. Respondent J noted fear of talking and suggested they feared that other learners would laugh at them. Respondent I also confirmed that they were afraid to answer because they had not done their homework or because they feared speaking a language other than their home language (Sesotho).

The fifth factor is immediacy. The majority of respondents (89%) agreed that immediacy does influence learner achievement in the classroom. Robinson's (2007) finding that relationships between educators and learners in the classroom does influence learner achievement is thus confirmed. Conversely, educators who do not encourage good relationships could be viewed as a barrier to learner achievement. During the interview educators indicated that they were not eager to develop personal relationships with learners because it could be misinterpreted as unprofessional.

Overall, respondents agree that classroom communication factors do influence learner achievement. Therefore, the findings validate the view that classroom communication can support learning but could also serve as a hindrance to learner performance. The following verbatim responses support the view regarding immediacy in the classroom.

*Good relationships do encourage learners to improve their marks. But it is difficult because learners take advantage. (Resp F)*

*I feel it is important, but it is more important to get the syllabus done. So yes, I try to have good relationships with my students. (Resp J)*

Based on these findings, it is suggested that educators should be made aware of the impact they have on the learning experience as communicators of relevant information.

Finally, teaching, as an interactive process, receives attention. Respondents are unanimous in their contention that teaching styles play a significant role in supporting learners to achieve good results. Alshare and Miller (2009) support this view that competence during instruction is vital in the overall educational experience of learners. While competent educators who are well versed in the LOLT can assist learners in acquiring knowledge and skills, those who are not proficient in the LOLT will not be able to impart information to learners effectively. This study has also confirmed Mji and Magato's (2006) view of teaching as an interactive process. The verbatim responses below support this approach, but they also note the challenges.

*Yes, it is but sometimes the learners do not want to participate in classroom discussions. They only want to listen. (Resp D)*

*Education is about interaction. Teaching requires that we communicate with our lives and get them involved in their own learning. I try to change the way I teach and get my learners to participate. (Resp H)*

The framework for educators to enhance communication has been designed based on the information obtained from the literature as well as the empirical investigation. Table 3 identifies the various communication factors, the barriers and a recommendation on how these specific aspects could be improved.

**Table 3:** Communication Framework for improved classroom communication.

Communication factor	Communication barrier	Recommended Improvement method
Verbal communication	Educators and learners are not fluent in the LOLT.	• The Department of Education (DoE) should provide developmental programmes that are language specific. • The DoE should partner with universities in their areas to provide short courses in English. • Subject Advisory Services – especially language specialists – should devise programmes to assist in this regard.
Non-verbal communication	Not all educators are aware of the impact of gestures when they convey information in the classroom.	• Educators should be made aware of the importance of non-verbal communication. • Communication workshops for all educators should be scheduled to enhance classroom communication – not only curricula workshops.
Verbal aggression	Educators demonstrating verbal aggression deter learners from wanting to openly express their opinions.	• Educators should be made aware of strategies that do not infringe on the rights of learners and adopt such strategies. • However, educators should be assertive to avoid problematic behaviour in the classroom.
Communication apprehension	Learners are afraid to express their views publicly.	• Educators should strive towards creating positive educational environments where learners are not apprehensive about asking questions. • Open two-way communication should be encouraged in the classroom.
Immediacy	A breakdown occurs in relationships between educators and learners.	• Educators should encourage open and honest communication with their learners.
Teaching as an interactive process	Educators are not comfortable about using a variety of teaching styles.	• Educators should receive refresher training in teaching methodology. • Departmental specialists should encourage educators to use different teaching styles to enhance their teaching.

An effective communication strategy and how this strategy is purposefully linked to organisational goals improves organisational success (Argenti, 2013). This argument can also apply to the classroom situation. A well-organised classroom that encourages interaction and an educator who has a goal in mind, will contribute towards the way learners master educational outcomes.

## 6 Conclusion and recommendations

The study was aimed at establishing how communication influences interaction between educators and learners in the classroom. It explored the extent to which miscommunication or a breakdown in communication between educators and learners could affect knowledge transfer. The qualitative data provides rich insights into the quantitative data. It was evident that factors such as communication apprehension and verbal aggression should be understood in order to enhance communication.

The study contributes to the existing body of knowledge by providing a framework on how educators can expand their knowledge to be effective communicators. Not only educators, but Department of Education officials can use this framework as a guideline to evaluate current communication practices, to identify good practices and key areas/schools where communication is ineffective and how these areas could possibly be improved.

The study was limited in the number of schools included in the investigation. Effective communication is only one significant aspect that influences learner achievement and only educators were included in this study. It is suggested that further research that includes learners' views on communication be conducted. Furthermore, an evaluation could be done of how effective communication practices are promoted between schools and the DoE as to ensure that schools are managed efficiently, and that important information is received timeously.

Communication between the new generation of learners and educators deserves to be addressed so that effective learning and teaching can occur. Awareness of effective communication strategies and how to enhance knowledge transfer can play a role in improving learning in schools. This study has shown that effective classroom communication is important, and the framework presented could serve as a means of improving classroom communication.

## Declarations

### Author contribution statement

Brenton. G. Fredericks: Conceived and designed the experiments; Performed the experiments; Analyzed and interpreted the data; Contributed reagents, materials, analysis tools or data; Wrote the paper.

Gregory Alexander: Conceived and designed the experiments; Analyzed and interpreted the data; Contributed reagents, materials, analysis tools or data.

### Funding statement

This work was supported by Central University of Technology and Fundamental Research Funds for Central Universities of the Central South University, South Africa.

### Data availability statement

Data included in article/supplementary material/referenced in article.

### Declaration of interests statement

The authors declare no conflict of interest.

### Additional information

No additional information is available for this paper.
